# Microbubble flows in superwettable fluidic channels†

**DOI:** 10.1039/c9ra04212a

**Published:** 2019-07-09

**Authors:** Mizuki Tenjimbayashi, Kotaro Doi, Masanobu Naito

**Affiliations:** Research and Services Division of Materials Data and Integrated System (MaDIS), National Institute for Materials Science (NIMS) 1-2-1 Sengen Tsukuba Ibaraki 305-0047 Japan TENJIMBAYASHI.Mizuki@nims.go.jp NAITO.Masanobu@nims.go.jp; Research Center for Structural Materials, National Institute for Materials Science (NIMS) 1-2-1 Sengen Tsukuba Ibaraki 305-0047 Japan

## Abstract

The control of bubble adhesion underwater is important for various applications, yet the dynamics under flow conditions are still to be unraveled. Herein, we observed the wetting dynamics of an underwater microbubble stream in superwettable channels. The flow of microbubbles was generated by integrating a microfluidic device with an electrochemical system. The microbubble motions were visualized *via* tracing the flow using a high-speed camera. We show that a vortex is generated in the air layer of the superaerophilic surface under laminar conditions and that the microbubbles are transported on the superaerophilic surface under turbulent conditions driven by the dynamic motion of the air film. Furthermore, microbubbles oscillated backward and forward on the superaerophobic surface under turbulent conditions. This investigation contributes to our understanding of the principles of drag reduction through wettability control and bubble flow.

## Abstract 2

Microbubble flows inside a superwettable channel revealed underwater superwetting phenomena under flow conditions, contributing to the understanding of real-world environmental wetting systems.

## Graphical abstract

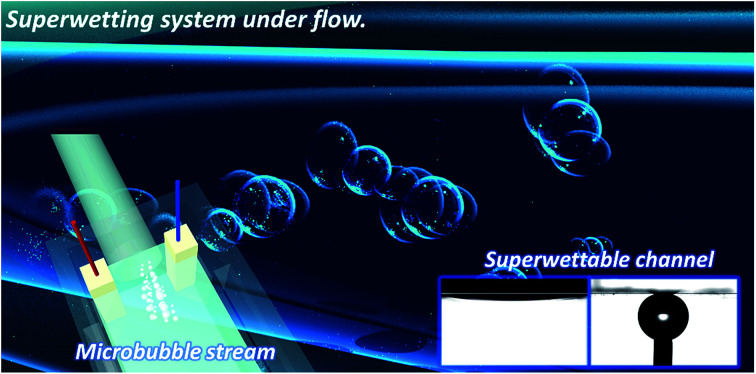

Nature offers us ideas for the design of materials with superwettability.^1^ In superwettable systems, the wetting of air underwater has generated interest recently.^2–6^ For example, penguin feathers are superaerophilic, with an air layer forming on the surface underwater, which allows penguins to swim in the sea with small amounts of drag.^2,3^ Inspired by this, researchers have theoretically and/or experimentally studied the influence of wettability on drag reduction underwater.^4–6^ In addition, fish scales are superaerophobic, which offers the idea of designing no-bubble adhesion electrodes that demonstrate high and stable oxygen evolution reaction performance.^7,8^ However, despite the development of superwettable materials for the controllable adhesion of air and/or bubbles underwater,^9^ the wetting dynamics of bubbles under flow conditions, which we must consider in real environments, have not been investigated.

Herein, we generated microbubble flows parallel to superwettable substrates inside a microfluidic device^10,11^ and studied the wetting dynamics through integrating an electrochemical setup^12^ with a microfluidic device, as shown in Fig. 1. The bubbles were formed through the electrolysis of water (see the ESI†). Two platinum plates were used: one as the working electrode and the other as the counter electrode. To increase the electrical conductivity, 2.0 mM K_2_SO_4_ was added to the water. The bath water–vapor interfacial tension, *γ*_LV_, was 71.9 ± 3.6 mN m^−1^ (*n* = 15) and the pH of the water was 7.8. We applied a current of ∼0.25 mA cm^−2^ to generate microbubbles with a diameter of 463.9 ± 245.1 μm (*n* = 120). The microfluidic device was generated using a 3D printer and connected to a water-flow generator (see the ESI† for the dimensions of the device). The microbubbles generated around the electrodes moved in the direction of the water flow and the coated substrates were placed parallel to the flow.

**Fig. 1:**
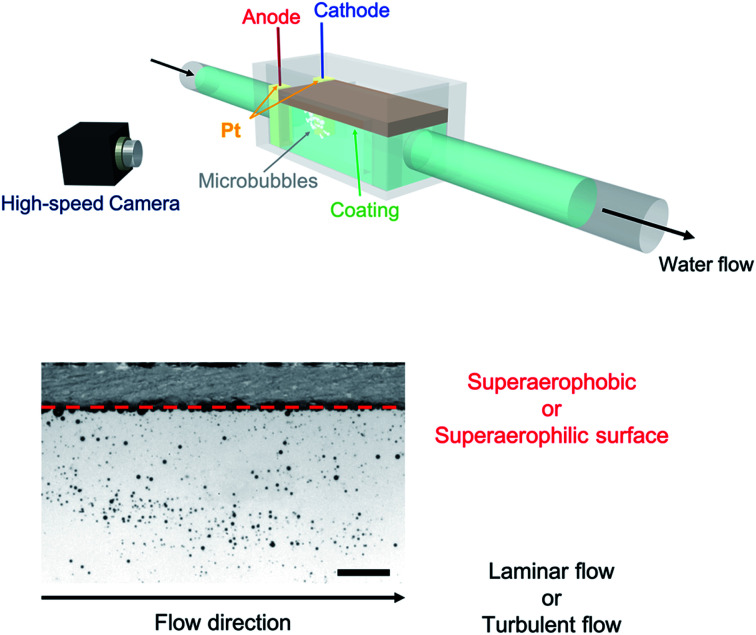
A schematic illustration of the microfluidic device with an electrochemical setup. We generated a flow of microbubbles and investigated the influences of coating wettability and flow type on the microbubbles dynamics *via* high speed camera observations. Scale bar: 10 mm.

We used the microbubbles as tracers and analyzed their flow as well as that of the water (*i.e.* microbubble image velocimetry), as shown in Fig. 2. We controlled the Reynolds number, Re = 4*Q*(π*Dν*)^−1^ (*Q* is the flow rate of the water, *D* is the tube diameter, and *ν* is the kinetic viscosity of the water). Laminar flow was obtained at Re = 79.21 and turbulent flow was obtained at Re = 396.06 (Fig. 2A). Under laminar flow conditions, the flow speed was nearly constant and the flow direction was close to perpendicular to the substrate (*φ* ≈ 0, where *φ* is the angle between the microbubble direction of movement and the width direction of the substrate) in all areas; this behavior was time-independent (Fig. 2B and C). Under turbulent flow conditions, the flow speed was not constant, and the flow direction was unstable (*φ* fluctuated between −180 and 180°) in all areas. We confirmed that the separation of flow did not occur, at least during the observation period, since the flow direction was parallel to the superwetting microfluidic device.

**Fig. 2:**
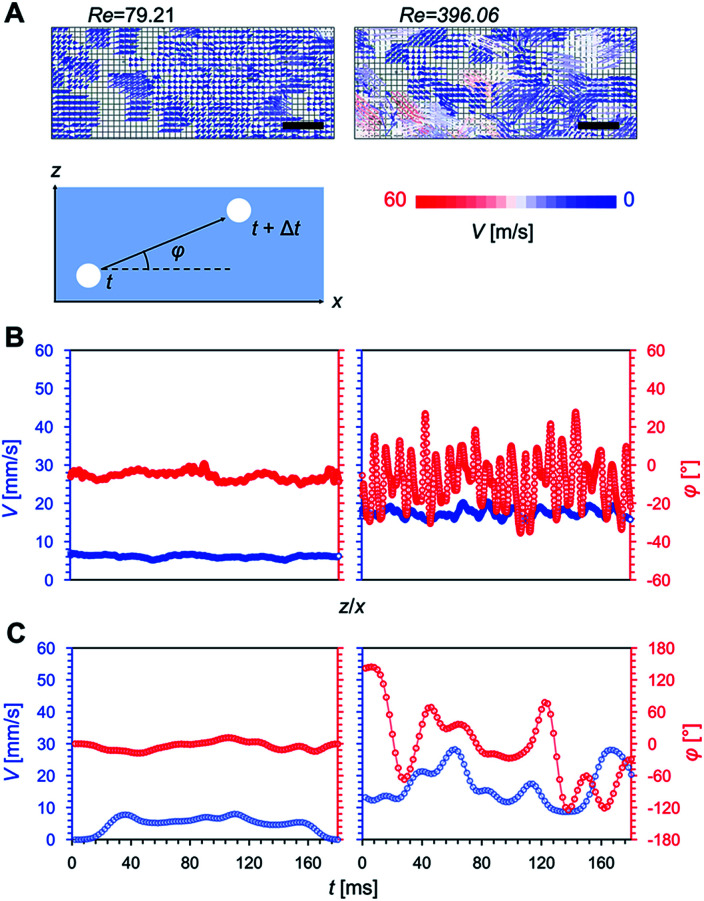
The flow conditions of the microbubbles. We created laminar and turbulent flows through altering the Reynolds number. (A) Flow velocimetry of the microbubbles under laminar (left) and turbulent (right) flow conditions; scale bar: 10 mm. (B) Velocity and flow direction profiles of microbubbles over the flow area under laminar (left) and turbulent (right) flow conditions. (C) Average velocity and flow direction fluctuations with time under laminar (left) and turbulent (right) flow conditions.

We then prepared substrate coatings with superaerophilicity and superaerophobicity. Superaerophilic substrates were fabricated according to our previous study.^12^ Concisely, a glass plate was dip-coated with a mixture of zinc oxide micro-tetrapod powder for surface roughening and polydimethylsiloxane for aerophilization. Superaerophobic surfaces were prepared through modifying a glass substrate with hydroxy groups using an aqueous potassium hydroxide solution.^13^ The wettability of the superaerophobic surfaces in relation to bubbles was confirmed *via* measuring the underwater bubble contact angle (*θ*); the results are shown in Fig. 3. We calculated the adhesion forces of bubbles, *F*_adh_ = π*l*^2^*γ*_LV_(1 + cos *θ*)/4,^14^ where *l* is the bubble–solid adhesion length. On the superaerophilic surface, the adhesion force was 3.2 × 10^3^ μN, and on the superaerophobic surface the force was 4.37 μN for 6 μL bubbles.

**Fig. 3:**
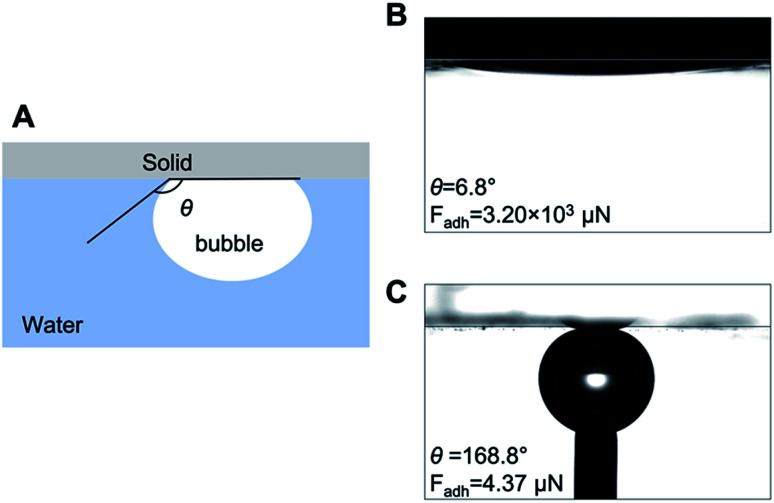
Wettability of the coatings. (A) schematic illustration of the measurement of the underwater air contact angle. The contact behavior of 6 μL microbubbles underwater on superaerophilic (B) and superaerophobic (C) surfaces.

In Fig. 4, we observed air film formation on superaerophilic surfaces under laminar and turbulent flow conditions. As we have previously shown, when microbubbles are vertically deposited on superaerophilic surfaces, a uniform air layer is formed.^10^ In the present study, under both laminar and turbulent flow conditions, a uniform air layer formed on the superaerophilic surfaces, but the air layers grew non-uniformly with the deposition of microbubbles owing to Rayleigh–Taylor instability^14^ (Fig. 4A and B). In all five independent observations, the shape of the air layer was non-uniform; thus, the flow of microbubbles influenced the shape of the air layer. However, bubbles with *l* = 4–7 mm formed on the surfaces under both laminar and turbulent flow conditions.

**Fig. 4:**
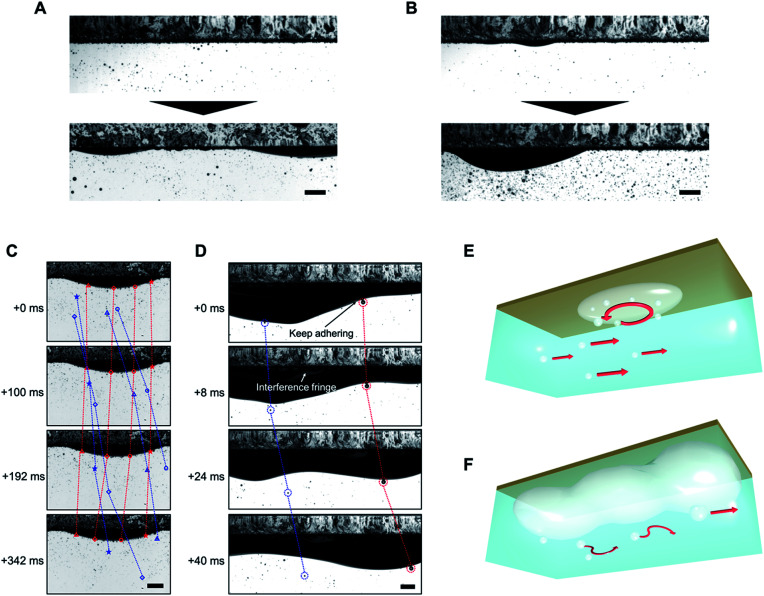
Microbubble deposition behavior on a superaerophilic surface under laminar (A) and turbulent (B) flow conditions. The top and bottom parts of the images are the initial and time-aged stages, respectively. (C) The vortex motion of microbubbles on deposited air films under laminar flow conditions. (D) Microbubbles transported on a superaerophilic surface under turbulent conditions driven by the dynamic motion of the air film. (E) and (F) Schematic representations of the microbubble behavior from (C) and (D), respectively. All scale bars: 10 mm.

After aging for 1000 s, a continuous air film formed on the superaerophilic surfaces under turbulent conditions. However, the shape was unstable and changed with time (Fig. 4D). In Fig. 4C and E, we observe the formation of a vortex on the hemispherical air film under laminar flow conditions (see Movie S1†). This phenomenon is interesting because under laminar flow conditions a vortex should not be generated (Fig. 2A); this cannot be explained using Bernoulli's theorem^15^ and the generation of a vortex suggests the separation of flows, which works to decrease flow resistance at the interface. Vortex generation may be due to the coalescence of microbubbles with the air layer, causing a change in the curvature of the hemispherical air film. This, in turn, would result in a change in the Laplace pressure of 2Δ*κγ*_LV_, where Δ*κ* is the change in curvature. There is a fluctuation in the vertical force torque to generate the vortex, and the force should be balanced by a Kutta–Joukowski force in the form of 2*γ*_LV_ d*κ*/d*t* ≈ *ρΓU*, where *ρ* is the density of flows, *Γ* is the vortex constant, and *U* is the velocity of the constant laminar flow.^16^

In Fig. 4D and F, we observe that microbubbles on the air film were transported as the shape of the air film dynamically changed to a wave-like nature; however, the microbubbles and air film did not coalesce (see Movie S2†). This indicates that a thin water layer exists between the microbubbles and the air film to prevent coalescence, whereas microbubbles are trapped on the air film by the buoyancy force of the microbubbles, which ≈(Δ*ρ*)*Ωg*, where Δ*ρ* is the difference in densities between a bubble and water, *Ω* is the volume of a microbubble, and *g* is gravitational acceleration.

We then observed the dynamics of the microbubbles on the superaerophobic surfaces (Fig. 5). As we have previously shown, when microbubbles are vertically deposited on superaerophobic surfaces, they are uniformly deposited on the surface and have a spherical shape.^12^ Under both laminar and turbulent flow conditions, microbubbles were deposited on the superaerophobic surfaces with spherical shapes but with non-uniform deposition (Fig. 5A and B). We then observed the motion of bubbles in contact with the superaerophobic surfaces. Under laminar flow conditions, microbubbles adhering to the surface moved in the direction of the flow (Fig. 5C and Movie S3†). In contrast, turbulent flow conditions caused the microbubbles to oscillate backward and forward (Fig. 5D and Movie S4†). The velocimetry profiles in Fig. 5E and F confirm that the bubble motion is linear in time under laminar flow, but it varies under turbulent flow (with the velocity periodically becoming negative). Despite the periodic negative velocity under turbulent flow conditions, the bubbles go forwards in the flow direction, which is not due to the laminar boundary but because the turbulent flow has more positive components than negative ones. This is because the length of positive motion under turbulent flow conditions increases with the size of the bubbles, obeying Newton's viscosity law.^17^ Thus, we confirmed that the motion of bubbles on superaerophobic surfaces is influenced by the flow conditions. The bubble motion distance on superaerophobic surfaces increased with bubble diameter.

**Fig. 5:**
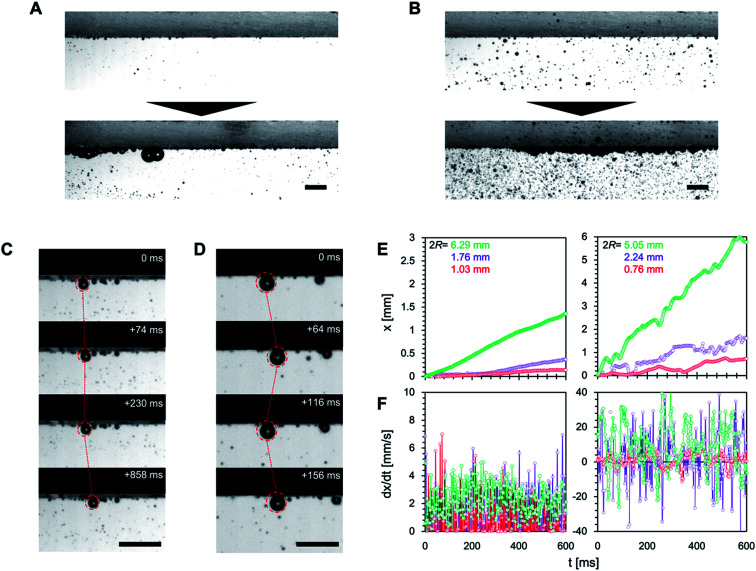
Microbubble deposition behavior on a superaerophobic surface under laminar (A) and turbulent conditions (B). The top and bottom parts of the images are the initial and time-aged stages, respectively. (C) Linear motion of the microbubbles on the surface under laminar flow. (D) The oscillating motion of microbubbles on the surface under turbulent flow. (E) Motion distance and (F) velocity analysis of microbubbles under laminar (left) and turbulent (right) conditions for different bubble diameters (2*R*). All scale bars: 10 mm.

## Conclusions

We investigated the wetting dynamics of microbubbles on superwettable surfaces under laminar and turbulent flow conditions. The microbubbles were non-uniformly deposited on the surfaces and the motion of the bubbles was influenced by the substrate wettability and the flow conditions. To discuss air adhesion on superwettable surfaces, the influence of flow must be considered, which strongly influences the hysteretic behavior of bubbles.^18^ For instance, vortex flow formation may be a crucial factor in deciding whether air can adhere to a solid or not; Taylor instability of the air layer dynamically changes the curvature and/or air/water contact area, strongly affecting the balance of solid–liquid–air interfacial energies. Moreover, the direct *in situ* observation of bubble flow will help create an understanding of the interfacial phenomena demonstrated by penguins, fish, and other swimmers in nature. In addition, this work may be helpful for understanding the influence of bubble leakage or cavitation on microfluidic systems.^19–21^

## Conflicts of interest

The authors declare no conflicts of interest associated with this work.

## Supplementary Material

RA-009-C9RA04212A-s001

RA-009-C9RA04212A-s002

RA-009-C9RA04212A-s003

RA-009-C9RA04212A-s004

RA-009-C9RA04212A-s005
