# Supplementary material for: Microbubble flows in superwettable fluidic channels
Source: RSC Adv. 2019 Jul 9;9(37):21220–4. doi: 10.1039/c9ra04212a (PMC9066019; doi:10.1039/c9ra04212a)
Supplement: RA-009-C9RA04212A-s001 [file RA-009-C9RA04212A-s001.pdf]

Electronic Supplementary Material (ESI) for RSC Advances.
This journal is © The Royal Society of Chemistry 2019

Supporting Information

Microbubble flows on superwettable fluidic channels.

Mizuki Tenjimbayashi,[†]* Kotaro Doi,[‡] Masanobu Naito[†]*
[†]Research and Services Division of Materials Data and Integrated System (MaDIS), National Institute for Materials Science (NIMS), 1-2-1 Sengen, Tsukuba Ibaraki, 305-0045, Japan.
[‡]Research Center for Structural Materials, National Institute for Materials Science (NIMS), 1-2-1 Sengen, Tsukuba Ibaraki, 305-0045, Japan.
*Correspondence to: TENJIMBAYASHI. Mizuki@nims.go.jp, NAITO.Masanobu@nims.go.jp

Contents

✓ *Gas generation by electrolysis*

✓ *Size of microfluidic device*

Gas generation by electrolysis

For gas generation, the electrolysis reactions of water under neutral conditions are:

$2H_2O + 2e^- \rightarrow H_2 + 2OH^-$ at the working electrode, and

$2H_2O \rightarrow O_2 + 4H^+ + 4e^-$ at the counter electrode. The density of bubbles can be controlled by alternating the current between the electrodes. The electrodes are placed inside the microfluidic device as shown in **Figure S1**.

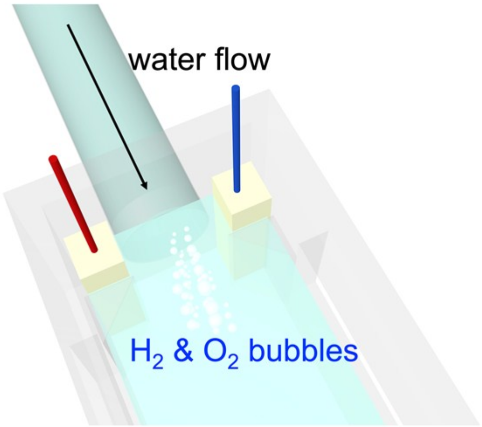

Figure S1. Schematic illustration of the electrode setup in the microfluidic device.

Dimensions of the microfluidic device

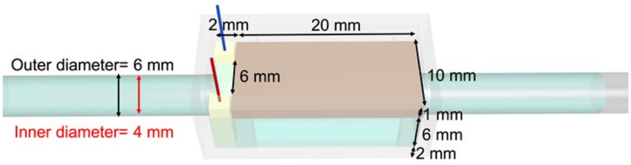

Figure S2. Schematic illustration of the microfluidic device. The dimensions of two platinum plates in contact with the water were 2 mm × 10 mm × 110 μm.
